# Large‐scale sequencing studies expand the known genetic architecture of Alzheimer's disease

**DOI:** 10.1002/dad2.12255

**Published:** 2021-12-31

**Authors:** Diane Xue, William S. Bush, Alan E. Renton, Edoardo A. Marcora, Joshua C. Bis, Brian W. Kunkle, The Alzheimer's Disease Sequencing Project, Eric Boerwinkle, Anita L. DeStefano, Lindsay Farrer, Alison Goate, Richard Mayeux, Margaret Pericak‐Vance, Gerard Schellenberg, Sudha Seshadri, Ellen Wijsman, Jonathan L. Haines, Elizabeth E. Blue

**Affiliations:** ^1^ Institute for Public Health Genetics University of Washington Seattle Washington USA; ^2^ Department of Population and Quantitative Health Sciences and Department of Genetics and Genome Sciences Case Western Reserve University Cleveland Ohio USA; ^3^ Cleveland Institute for Computational Biology Cleveland Ohio USA; ^4^ Department of Genetics and Genomic Sciences Nash Family Department of Neuroscience and Ronald M. Loeb Center for Alzheimer's Disease Icahn School of Medicine at Mount Sinai New York New York USA; ^5^ Cardiovascular Health Research Unit Department of Medicine University of Washington Seattle Washington USA; ^6^ The John P. Hussman Institute for Human Genomics University of Miami Miami Florida USA; ^7^ Dr. John T Macdonald Foundation Department of Human Genetics Miller School of Medicine University of Miami Miami Florida USA; ^8^ Human Genome Sequencing Center Department of Molecular and Human Genetics Baylor College of Medicine Houston Texas USA; ^9^ School of Public Health University of Texas Health Science Center at Houston Houston Texas USA; ^10^ Department of Biostatistics Boston University Boston Massachusetts USA; ^11^ Department of Neurology Boston University Boston Massachusetts USA; ^12^ Division of Biomedical Genetics Department of Medicine Department of Epidemiology and Department of Ophthalmology Boston University Boston Massachusetts USA; ^13^ Department of Genetics and Genomics Sciences and Friedman Brain Institute Mount Sinai School of Medicine New York New York USA; ^14^ Taub Institute for Research on Alzheimer's Disease and the Aging Brain Gertrude H. Sergievsky Center Department of Neurology Department of Psychiatry and Epidemiology Columbia University New York New York USA; ^15^ Penn Neurodegeneration Genomics Center Department of Pathology and Laboratory Medicine University of Pennsylvania Philadelphia Pennsylvania USA; ^16^ Glenn Biggs Institute for Alzheimer's & Neurodegenerative Diseases and Department of Neurology University of Texas Health Science Center San Antonio Texas USA; ^17^ Division of Medical Genetics University of Washington Seattle Washington USA; ^18^ Department of Biostatistics University of Washington Seattle Washington USA

**Keywords:** Alzheimer's disease, genetic architecture, genome, networks, pathways

## Abstract

**Introduction:**

Genes implicated by genome‐wide association studies and family‐based studies of Alzheimer's disease (AD) are largely discordant. We hypothesized that genes identified by sequencing studies like the Alzheimer's Disease Sequencing Project (ADSP) may bridge this gap and highlight shared biological mechanisms.

**Methods:**

We performed structured literature review of genes prioritized by ADSP studies, genes underlying familial dementias, and genes nominated by genome‐wide association studies. Gene set enrichment analyses of each list identified enriched pathways.

**Results:**

The genes prioritized by the ADSP, familial dementia studies, and genome‐wide association studies minimally overlapped. Each gene set identified dozens of enriched pathways, several of which were shared (e.g., regulation of amyloid beta clearance).

**Discussion:**

Alternative study designs provide unique insights into AD genetics. Shared pathways enriched by different genes highlight their relevance to AD pathogenesis, while the patterns of pathway enrichment unique to each gene set provide additional targets for functional studies.

## 1 BACKGROUND

Alzheimer's disease (AD) is the leading cause of dementia in the United States, estimated to affect 5.8 million Americans in 2020.^1^ AD is a complex and highly heritable trait^2^ for which there is no efficacious treatment. Drug targets supported by human genetic evidence are much more likely to be approved by the Food and Drug Administration for therapeutic use,^3^ demonstrating the need for continued genetics research into AD and an improved understanding of the biological processes underlying the disease.

The known genetic architecture of AD implicates causal and risk variants at dozens of loci.^4^ Family studies have illustrated that rare early‐onset autosomal dominant AD (ADAD) can be caused by highly penetrant variants in *APP*,^5^ *PSEN1*,^6^ and *PSEN2*.^7^ Although these autosomal dominant variants explain the cause of AD in < 1% of cases,^8^ their discovery provided a direct link between AD genetics and pathogenesis through rare coding changes^9^ in genes underlying the generation of amyloid beta (Aβ), a neuropathological hallmark of AD.^10^ The apolipoprotein E (*APOE*) ε2 and ε4 alleles defined by two missense variants were first associated with AD in family studies and underlie the strongest signal across genome‐wide association studies (GWAS) of AD.^11^, ^12^, ^13^, ^14^ Rare variant association studies have also identified protein coding changes associated with AD,^15^ though many of these studies have been restricted to analyses of known variants (e.g., *ABI3*, *PLCG2* ^16^) or small samples of whole exome sequence (WES) data (e.g., *AKAP9*,^17^ *TREM2* ^18^). Large GWAS of common variants have implicated dozens of loci but do not implicate the ADAD genes.^13^, ^19^ Many of the AD GWAS loci are intergenic, and the specific genes influencing AD risk and pathogenesis within those loci are mostly unresolved.^19^ The genes implicated by family studies and GWAS approaches are largely discordant, influenced in part by their study design: family‐based studies have better power to detect rare variants with large effect sizes, while GWAS are better powered to identify common variants associated with modest effect sizes but typically representing a single ancestry. Large‐scale sequencing efforts like the Alzheimer's Disease Sequencing Project (ADSP^20^) may resolve the link between GWAS locus and functional variation by directly testing sequence variation rather than genetic markers or imputed genotypes. We hypothesize that the genes implicated in AD risk by these different analytical strategies may represent shared biological pathways.

Instead of relying on a single gene's story, pathway analyses identify enrichment in biological functions among members of a gene set.^21^ These approaches have connected genes near GWAS loci to biological processes that may influence AD pathogenesis.^12^, ^13^ Pathway analyses are frequently restricted to the genes or loci implicated by a single study rather than the field as a whole and may miss connections with genes implicated by alternative study designs. If the support for a given pathway is strong, one could imagine targeting therapeutic interventions or treatments to those pathways, as opposed to a single gene.^20^

Here, we summarize the genes implicated by the ADSP Discovery Phase publications and place them into the larger context of AD genetics. We compare the genes implicated by the ADSP with genes underlying familial dementias and genes prioritized in a recent meta‐analysis of AD GWAS representing > 90,000 subjects (35,274 cases and 59,163 controls)^13^ or an AD genetics literature review.^22^ Gene set enrichment analyses identify biological processes implicated by these three different avenues of AD genetics research. We hypothesize that the genes implicated by the ADSP will provide greater resolution within established AD pathways and may implicate new pathways relevant to disease.

**RESEARCH IN CONTEXT**

1. **Systematic review**: Genes implicated by the Alzheimer's Disease Sequencing Project (ADSP) underwent a literature review to identify prior evidence for a relationship to Alzheimer's disease (AD). Gene set enrichment analyses compared the pathways implicated by the subset of ADSP genes with independent support to those implicated in familial dementias or genome‐wide or association studies.
2. **Interpretation**: While the ADSP, familial dementia, and genome‐wide association study gene sets are largely discordant, they are enriched in genes representing similar biological pathways (e.g., regulation of amyloid beta clearance). Gene set–specific pathways highlight the utility of alternative strategies for identifying genetic variation influencing AD risk and pathogenesis.
3. **Future directions**: The genes and pathways highlighted here present targets for further functional and neuropathological studies, as well as pathway‐specific genetic risk scores. Increasingly diverse study populations and approaches within AD research are expected to identify novel genes that may provide support for these pathways or nominate others.

**HIGHLIGHT**

- Exome and genome‐based Alzheimer's disease studies nominate novel genes/pathways
- Common and rare variant studies support genes within several biological pathways
- *APOE*, *AKAP9*, *MAPT*, *ABCA7*, *CSF1R*, and *TREM2* contributed to the most ADSP pathways
- Functional studies support most Alzheimer's Disease Sequencing Project genes

## 2 MATERIALS AND METHODS

### 2.1 AD GWAS gene set

The curated AD GWAS gene list includes the genes summarized in two recent publications: a literature review of sporadic or late‐onset AD risk loci implicated by linkage and/or association studies^22^ (N = 16 studies, sample size = 40–113,600) and a meta‐analysis of 94,437 clinically diagnosed AD subjects.^13^ These two references represent samples with European ancestry and do not include stratified analyses or studies of biomarkers, endophenotypes, or family history of dementia. Most of these associations involve single‐variant tests of common, non‐coding markers, although a handful of rare variant studies were included.^22^ The 31 genes extracted from the review paper were restricted to a single gene at each locus prioritized by the authors of the review. The meta‐analysis combined evidence from coding changes, gene expression, pathway analyses, and clinical expression to nominate 53 candidate genes across 24 genome‐wide significant loci, including most of the genes extracted from the review paper (17/31 = 55%).

### 2.2 Familial dementia gene set

Genes underlying AD, dementias which can clinically mimic AD such as frontotemporal dementia (MIM:600274), and distinct dementias such as leukoencephalopathy with vanishing white matter (MIM:603896) were extracted from a clinical neurodegenerative disease gene panel followed by literature review^9^ (Table S1 in supporting information). *C9ORF72*, a gene underlying frontotemporal dementia^23^ previously associated with AD,^24^ was added to complete the familial dementia gene set (N = 36). Most of these gene–phenotype relationships were identified by the co‐segregation of the phenotype with rare coding changes in small, family‐based studies.

### 2.3 The AD sequencing project gene set

The ADSP, supported jointly by the National Institute on Aging and the National Human Genome Research Institute, gathers and analyzes WES and whole genome sequence (WGS) data to detect novel AD risk variants.^20^ The ADSP Discovery Phase was a collaboration between the Alzheimer's Disease Genetics Consortium and the Cohorts for Heart and Aging Research in Genomic Epidemiology Consortium.^20^ The ADSP Discovery Phase produced eight gene‐discovery publications: three using WGS data from 582 individuals from 111 families with either European American or Caribbean Hispanic ancestry^9^, ^25^, ^26^ and five publications based upon WES representing > 10,000 subjects with primarily non‐Hispanic White ancestry.^27^, ^28^, ^29^, ^30^, ^31^ Sample sizes within these studies range from 5740 cases and 5096 controls with European American or Caribbean Hispanic ancestry^27^ to 164 cases and 33 controls within 42 families with non‐Hispanic European ancestry.^25^

Genes with evidence for a relationship with AD risk were extracted from ADSP Discovery Phase publications using permissive filters. Genes from the family‐based WGS studies were extracted if they met one or more of the following conditions: (1) variation in genes belonging to the familial dementia gene set which either was previously reported as pathogenic or co‐segregated with AD in at least one family within the ADSP, (2) variation within genes from the AD GWAS gene set with either evidence for association with AD or co‐segregation in 2+ families, or (3) variation co‐segregating with AD in 2+ families within a multi‐family linkage region. Genes from the ADSP WES studies were extracted if their support met at least one of the following conditions: (1) variation with exome‐wide significant evidence of association at the variant or gene level or (2) variation includes rare coding variants in 10+ cases and no controls. All gene names were verified using the multi‐symbol checker developed by the HUGO Gene Nomenclature Committee (HGNC) multi‐symbol checker.

Genes meeting these permissive criteria underwent structured literature reviews by two investigators, and the two earliest references supporting a link between AD and the gene were recorded where available. First, we searched for “gene” AND “Alzheimer” in PubMed and reviewed the entries from oldest to newest. We then reviewed the Online Mendelian Inheritance in Man (OMIM^32^) for each gene for a connection to AD. Finally, we searched for ‘“gene” and “Alzheimer”’ and reviewed the first two pages of matches for references supporting the gene to AD link using https://scholar.google.com (last accessed March 22, 2021). Papers were included as evidence of a connection between the gene and AD if the gene was associated with AD‐specific changes in genotype or gene expression, or AD‐specific endophenotypes, pathology, or biomarkers in humans or animal models at a study‐wide statistical significance level. References were excluded from the review if the research was an abstract for a conference, part of a dissertation, not published in English, or linked only to an AD risk factor (e.g., aging). Genes with at least one external publication supporting a link to AD were included in the ADSP‐derived gene set (ADSP+) used for pathway analysis.

### 2.4 Gene set enrichment analysis

Gene sets were provided to STRING‐db (v11.0^33^) to test for protein–protein interaction (PPI) enrichment using most default parameter settings but dropping text mining of PubMed abstracts and neighborhood of the genome as sources of interaction. Genes in our gene sets have been published together by definition, and the gene list derived from GWAS provided multiple gene candidates at a single locus, both of which would bias results if text mining or gene neighborhood were allowed as a source. Tests for PPI applied a significance threshold of *P* < .05. Gene set enrichment analyses were performed using the eXploring Genomic Relations for enhanced interpretation (XGR) software^34^ to identify significantly enriched pathways among familial dementia, GWAS, and ADSP+ gene sets. Each gene set was tested for enrichment in Gene Ontology (GO) biological processes using a hypergeometric test accounting for ontological structure and redundant pathways, excluding gene sets with fewer than two genes, and using all human genes as the reference. The significance threshold was set to a false discovery rate (FDR) < 0.05.^33^ Using the GeneOverlap R package (v3.12),^35^ Fisher's exact test was used to test for evidence of significant overlap between genes driving the enrichment of each pair of pathways, with a significance threshold of *P* < .05.

## 3 RESULTS

### 3.1 ADSP+, AD GWAS, and familial dementia gene sets

**FIGURE 1:**
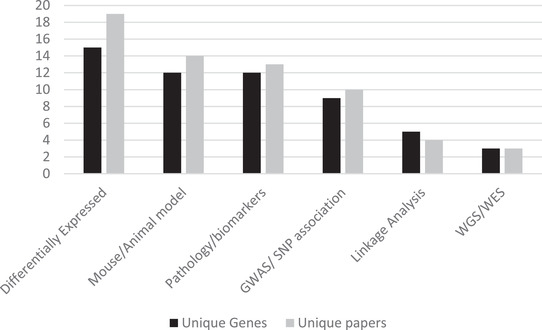
Sources of literature support for Alzheimer's Disease Sequencing Project (ADSP) Discovery Phase candidate genes. Differentially expressed genes (N = 15) include *ABCD4, CELSR1, GAS2L2, GIMAP2, GPAA1, GRN, KANSL3, NPC1, QRICH2, SCFD1, SCN4A, SLC24A3, SPHK2, STAB1, SUV420H1/KMT5B*. Mouse/animal model genes (N = 12) include *ABCA7, CELSR1, CHMP2B, CSF1R, DDR2, GTSE1, HTR3A, NSF, TMEM87A, TREM2, TTC3, UBAP2*. Pathology/biomarkers genes (N = 12) include *APOE, CASP7, CBLC, CHMP2B, DDR2, KCNK13, MAPT, NOTCH3, OPRL1, PINX1, STAB1, ZNF655*. Genome‐wide association study (GWAS)/single nucleotide polymorphism (SNP) association genes (N = 9) include *ABCA7, APOE, ARSA, CASP7, FERMT2, L3MBTL2, MS4A6A, NPC1, PILRA*. Linkage analysis genes (N = 5) include *ABCD4, CSF1R, NOTCH3, OR8G5, TTC3*. Whole genome sequence (WGS)/Whole exome sequence (WES) genes (N = 3) include *AKAP9, BCAM, CBLC*. Complete details available in Table S1 in supporting information

Across the eight ADSP Discovery Phase studies,^9^, ^25^, ^26^, ^27^, ^28^, ^29^, ^30^, ^31^ 64 genes met our permissive criteria (Table S2 in supporting information). Independent support for a link to AD was identified for the majority of these genes (43/64, 67%), defining the ADSP+ gene set (Table 1). Most of these genes were reported in a single ADSP Discovery Phase study, though *TREM2* appeared in four studies.^27^, ^28^, ^30^, ^31^ Much of the literature support for the ADSP+ genes come from functional studies, rather than statistical associations (Figure 1, Table S2). Studies identifying genes differentially expressed in AD supported the highest number of genes (15 genes), closely followed by studies of genes related to changes in AD pathology (12 genes) or animal models (12 genes), GWAS or single nucleotide polymorphism (SNP) association studies (9 genes), linkage analyses (5 genes), and WES/WGS studies (3 genes). The relatively sparse support from WES/WGS studies almost certainly reflects the relative scarcity of large sequencing studies of AD prior to the ADSP.

**TABLE 1:** Origins of genes belonging to the ADSP+, familial dementia, and GWAS gene sets

Gene Set	Source	Data	Genes
ADSP+	Bis et al. (2020)^27^	ADSP WES	*ABCA7, APOE, BCAM, CBLC, GAS2L2, MS4A6A, OPRL1, PILRA, TREM2, ZNF655*
	Ma et al. (2019)^28^	ADSP WES	*GPAA1, MAPT, NSF, OR8G5, SLC24A3, TREM2*
	Patel et al. (2019)^29^	ADSP WES	*ABCD4, CELSR1, GIMAP2, GTSE1, L3MBTL2, NOTCH3, QRICH2, SCFD1, SPHK2, SUV420H1, UBAP2*
	Tosto et al. (2019)^30^	ADSP WES	*PINX1, TREM2*
	Zhang et al. (2019)^31^	ADSP WES	*CASP7, HTR3A, KANSL3, KCNK13, NPC1, SCN4A, STAB1, TMEM87A, TREM2*
	Beecham et al. (2018)^25^	ADSP WGS	*DDR2, FERMT2, TTC3*
	Blue et al. (2018)^9^	ADSP WGS	*ARSA, CHMP2B, CSF1R, GRN*
	Vardarajan et al. (2018)^26^	ADSP WGS	*AKAP9*
Familial dementia	Dementia gene panel^9^	Clinical test	*APOE, APP, ARSA, ATP13A2, C9orf72, CHCHD10, CHMP2B, CSF1R, DNMT1, EIF2B1, EIF2B2, EIF2B3, EIF2B4, EIF2B5, FUS, GALC, GRN, HEXA, ITM2B, LMNB1, MAPT, NOTCH3, NPC1, NPC2, PDGFB, PDGFRB, PRNP, PSEN1, PSEN2, SLC20A2, SLC25A12, TARDBP, TBP, TREM2, TYROBP, VCP*
GWAS	Kunkle *et al*. (2019),^13^ Figure 2	GWAS and annotation	*ABCA7, ACP2, ADAM10, ADAMTS1, AGFG2, ARHGAP45 (HMHA1), BIN1, C1QTNF4, C4A, CASS4, CD2AP, CD55, CELF1, CLU, CNN2, CR1, ECHDC3, EED, EPHB4, FAM131B, GAL3ST4, GPSM3, HLA‐DPA1, HLA‐DQA1, HLA‐DRA, HLA‐DRB1, HLA‐DRB5, INPP5D, IQCK, MAF, MAP11 (C7orf43), MS4A4A, MS4A6A, MS4A7, MTCH2, NYAP1, NDUFS3, NUP160, PICALM, PILRA, PSMB8, PSMB9, PSMC3, PSMC5, PTK2B, RIN3, SORL1, SPI1, STYX, TREM2, WDR18, WWOX, YOD1, ZKSCAN1*
	Naj et al. (2017)^22^ review of 16 publications, Table 2	GWAS and linkage analysis	*ABCA7, ACE, APOE, APP, BIN1, CASS4, CD2AP, CD33, CELF1, CLU, CR1, DSG2, EPHA1, FERMT2, HLA‐DRB1, INPP5D, MEF2C, MS4A gene cluster, NME8, PICALM, PLD3, PTK2B, RIN3, SLC24A4, SORL1, TREM2, TREML2, TRIP4, ZCWPW1*

Abbreviations: ADSP, Alzheimer's Disease Sequencing Project; GWAS, genome‐wide association study; WES, whole exome sequence; WGS, whole genome sequence.

The GWAS gene set includes 70 genes derived from 17 publications (Table 1).^13^, ^22^ Six of the GWAS genes (9%) overlap with the ADSP+ gene set: *ABCA7*, *APOE*, *FERMT2*, *MS4A6A*, *PILRA*, and *TREM2*. The familial dementia gene set includes 36 genes derived from a clinical testing panel for neurodegenerative disease supplemented with literature review (Table 1).^9^ Nine of the familial dementia genes (25%) overlap with the ADSP+ gene set: *APOE, ARSA, CHMPB, CSF1R, GRN, MAPT, NOTCH3, NPC1*, and *TREM2*. The familial dementia and AD GWAS gene sets are largely discordant, sharing only *APOE*, *APP*, and *TREM2*.

### 3.2 Gene set enrichment analysis

The genes within the ADSP+ gene list exhibit significant evidence of interaction and represent many biological pathways. The ADSP+ genes exhibit significant PPI enrichment (*P *= 8.36E‐03), with seven PPI edges observed between 43 nodes when two edges were expected under the null hypothesis. These edges form four clusters: (1) *CSF1R* is co‐expressed with *TREM2*, *MS4A6A*, and *STAB1* with the latter two also co‐expressed with each other; (2) *ABCA7* and *ABCAD4* are co‐expressed and associated with each other in a curated database; as are (3) *ARSA* and *GRN*; while (4) *NSF* and *SCFD1* are co‐expressed, associated in a curated database, and their proteins physically interact as measured with biochemical data.^33^ XGR analyses of the ADSP+ genes identified 45 significantly enriched biological processes (Table 2). The top two ADSP+ pathways, regulation of Aβ clearance (GO:1900221, FDR = 2.60E‐05) and cholesterol efflux (GO:0033344, FDR = 9.00E‐05), have much stronger support than the remaining 43 pathways (0.05 > FDR > 0.005). Both the familial dementia gene set (FDR = 8.80E‐05; *APOE, TREM2*) and the GWAS gene set (FDR = 2.70E‐07; *ABCA7, APOE, CLU, TREM2*) were significantly enriched in genes belonging to the regulation of Aβ clearance (GO:1900221) pathway. The familial dementia gene set is also enriched in genes belonging to the cholesterol efflux pathway (GO:0033344; FDR = 1.00E‐05; *ABCA7, APOE, NPC1, NPC2*), while the AD GWAS gene set is not (FDR > 0.05).

**TABLE 2:** Pathways identified by ADSP+ gene set enrichment analysis

GO ID	Term Name	FDR	Genes
GO:1900221	Regulation of amyloid beta clearance	2.60E‐05	*ABCA7, APOE, TREM2*
GO:0033344	Cholesterol efflux	9.00E‐05	*ABCA7, APOE, NPC1*
GO:0051651	Maintenance of location in cell	2.60E‐03	*AKAP9, APOE, GPAA1*
GO:0031116	Positive regulation of microtubule polymerization	2.60E‐03	*AKAP9, MAPT*
GO:0016242	Negative regulation of macroautophagy	2.60E‐03	*NPC1, SCFD1*
GO:0070374	Positive regulation of ERK1 and ERK2 cascade	2.70E‐03	*ABCA7, APOE, CSF1R, TREM2*
GO:0019068	Virion assembly	2.70E‐03	*APOE, CHMP2B*
GO:0030316	Osteoclast differentiation	2.70E‐03	*CSF1R, TREM2*
GO:0007613	Memory	2.90E‐03	*ABCA7, APOE, MAPT*
GO:0007080	Mitotic metaphase plate congression	3.00E‐03	*CHMP2B, PINX1*
GO:0007160	Cell‐matrix adhesion	3.10E‐03	*BCAM, DDR2, FERMT2*
GO:0061024	Membrane organization	3.90E‐03	*ABCA7, APOE, CHMP2B, NPC1, NSF, SCFD1, TREM2*
GO:0048844	Artery morphogenesis	3.90E‐03	*APOE, NOTCH3*
GO:0048278	Vesicle docking	4.30E‐03	*NSF, SCFD1*
GO:0034765	Regulation of ion transmembrane transport	4.60E‐03	*AKAP9, HTR3A, KCNK13, OPRL1, SCN4A*
GO:1900182	Positive regulation of protein localization to nucleus	4.60E‐03	*GTSE1, PINX1*
GO:0010948	Negative regulation of cell cycle process	4.90E‐03	*GTSE1, L3MBTL2, PINX1, ZNF655*
GO:0006813	Potassium ion transport	4.90E‐03	*KCNK13, NSF, SLC24A3*
GO:1902749	Regulation of cell cycle G2/M phase transition	5.10E‐03	*AKAP9, GTSE1, PINX1*
GO:0043407	Negative regulation of MAP kinase activity	5.80E‐03	*APOE, CBLC*
GO:0035725	Sodium ion transmembrane transport	7.90E‐03	*SCN4A, SLC24A3*
GO:0032414	Positive regulation of ion transmembrane transporter activity	9.60E‐03	*AKAP9, HTR3A*
GO:0007267	Cell‐cell signaling	1.00E‐02	*AKAP9, APOE, CELSR1, FERMT2, HTR3A, MAPT, STAB1*
GO:0050848	Regulation of calcium‐mediated signaling	1.00E‐02	*MAPT, TREM2*
GO:0042327	Positive regulation of phosphorylation	1.10E‐02	*ABCA7, AKAP9, APOE, CSF1R, DDR2, MAPT, TREM2*
GO:0051656	Establishment of organelle localization	1.10E‐02	*CHMP2B, MAPT, NSF, PINX1, SCFD1*
GO:0006664	Glycolipid metabolic process	1.10E‐02	*ARSA, GPAA1*
GO:0042391	Regulation of membrane potential	1.30E‐02	*AKAP9, HTR3A, KCNK13, MAPT, SCN4A*
GO:0006897	Endocytosis	1.40E‐02	*ABCA7, APOE, NPC1, STAB1, TREM2*
GO:0006475	Internal protein amino acid acetylation	1.40E‐02	*KANSL3, MAPT*
GO:0043269	Regulation of ion transport	1.60E‐02	*ABCA7, AKAP9, APOE, HTR3A, KCNK13, OPRL1, SCN4A*
GO:0051348	Negative regulation of transferase activity	1.60E‐02	*APOE, CBLC, MAPT, PINX1*
GO:0022604	Regulation of cell morphogenesis	2.00E‐02	*APOE, CSF1R, FERMT2, MAPT*
GO:0007626	Locomotory behavior	3.10E‐02	*APOE, CELSR1, NPC1*
GO:0040017	Positive regulation of locomotion	3.20E‐02	*CHMP2B, CSF1R, DDR2, GRN, GTSE1*
GO:0006643	Membrane lipid metabolic process	3.20E‐02	*ARSA, GPAA1, SPHK2*
GO:0050795	Regulation of behavior	3.30E‐02	*APOE, OPRL1*
GO:0018108	Peptidyl‐tyrosine phosphorylation	3.50E‐02	*CSF1R, DDR2*
GO:0051174	Regulation of phosphorus metabolic process	4.40E‐02	*ABCA7, AKAP9, APOE, CBLC, CSF1R, DDR2, MAPT, TREM2*
GO:0061097	Regulation of protein tyrosine kinase activity	4.50E‐02	*CBLC, CSF1R*
GO:0016192	Vesicle‐mediated transport	4.70E‐02	*ABCA7, APOE, ARSA, CHMP2B, GRN, NPC1, NSF, SCFD1, STAB1, TMEM87A, TREM2*
GO:0006644	Phospholipid metabolic process	4.90E‐02	*CSF1R, GPAA1, SPHK2*
GO:0006874	Cellular calcium ion homeostasis	4.90E‐02	*APOE, OPRL1, SLC24A3*
GO:0099177	Regulation of trans‐synaptic signaling	4.90E‐02	*AKAP9, APOE, MAPT*
GO:0006942	Regulation of striated muscle contraction	4.90E‐02	*AKAP9, SCN4A*

Abbreviations: ADSP, Alzheimer's Disease Sequencing Project; FDR, false discovery rate; GO, Gene Ontology.

Note: Significant results were defined as with FDR < 0.05.

The intersection of pathways enriched by ADSP+ genes with those enriched by the familial dementia genes (N = 116, Table S3 in supporting information) and AD GWAS genes (N = 102, Table S4 in supporting information) provides insight into the genetic architecture of AD. Nine pathways are enriched by both the ADSP+ and familial dementia genes, seven are enriched by both the ADSP+ and AD GWAS genes, and four are enriched in analyses of all three gene sets (Table 3). For some of these shared pathways, the ADSP+ gene set contributes unique genes absent from the familial dementia and AD GWAS sets, fleshing out pathways previously implicated in AD. In addition to *ABCA7*, *APOE*, *NPC1*, and *TREM2*, endocytosis (GO:0006897) is also supported by the ADSP+ gene *STAB1*. The ADSP+ genes also add *AKAP9* and *DDR2* to the list of genes implicating regulation of phosphorous metabolic process (GO:0051174) and *CBLC* (in the *APOE* region) to regulation of protein tyrosine kinase activity (GO:0061097).

**TABLE 3:** Pathways significantly enriched in genes from ADSP+ gene list that overlap with those enriched in the familial dementia gene list, the GWAS gene list, or both

		ADSP+ gene set		Familial dementia gene set		GWAS gene set	
GO ID	GO term name	FDR	Genes	FDR	Genes	FDR	Genes
GO:1900221	Regulation of amyloid beta clearance	2.60E‐05	*ABCA7, APOE, TREM2*	8.80E‐05	*APOE, TREM2*	2.70E‐07	*ABCA7, APOE, CLU, TREM2*
GO:0006897	Endocytosis	1.40E‐02	*ABCA7, APOE, NPC1*, ***STAB1*** *, TREM2*	7.60E‐03	*APOE, APP, C9orf72, NPC1, TREM2*	3.20E‐03	*ABCA7, APOE, APP, BIN1, PICALM, RIN3, SORL1, TREM2*
GO:0051174	Regulation of phosphorus metabolic process	4.40E‐02	*ABCA7*, ***AKAP9*** *, APOE, CBLC, CSF1R*, ***DDR2*** *, MAPT, TREM2*	8.70E‐03	*APOE, APP, C9orf72, CSF1R, MAPT, PDGFB, PDGFRB, PRNP, PSEN1, SLC25A12, TARDBP, TREM2, VCP*	2.80E‐02	*ABCA7, ACE, APOE, APP, CASS4, CLU, EPHA1, MEF2C, PTK2B, SORL1, STYX, TREM2*
GO:0061097	Regulation of protein tyrosine kinase activity	4.50E‐02	***CBLC*** *, CSF1R*	3.20E‐06	*APP, CSF1R, PDGFB, PRNP, PSEN1*	3.10E‐03	*ACE, APP, CASS4*
GO:0022604	Regulation of cell morphogenesis	2.00E‐02	*APOE, CSF1R, FERMT2, MAPT*	NA	NA	2.20E‐02	*ADAM10, APOE, CASS4, FERMT2, PTK2B*
GO:0099177	Regulation of trans‐synaptic signaling	4.90E‐02	***AKAP9*** *, APOE, MAPT*	NA	NA	1.70E‐02	*APOE, APP, MEF2C, PSMC5, PTK2B*
GO:0006874	Cellular calcium ion homeostasis	4.90E‐02	*APOE*, ***OPRL1***, ***SLC24A3***	NA	NA	3.90E‐02	*APOE, APP, CD55, PTK2B, SLC24A4*
GO:0033344	Cholesterol efflux	9.00E‐05	*ABCA7, APOE, NPC1*	1.00E‐05	*APOE, NPC1, NPC2*	NA	NA
GO:0070374	Positive regulation of ERK1 and ERK2 cascade	2.70E‐03	*ABCA7, APOE, CSF1R, TREM2*	1.60E‐05	*APOE, APP, CSF1R, PDGFB, PDGFRB, TREM2*	NA	NA
GO:0019068	Virion assembly	2.70E‐03	*APOE, CHMP2B*	7.90E‐04	*APOE, CHMP2B*	NA	NA
GO:0048844	Artery morphogenesis	3.90E‐03	*APOE, NOTCH3*	1.80E‐04	*APOE, NOTCH3, PDGFRB*	NA	NA
GO:0042391	Regulation of membrane potential	1.30E‐02	*AKAP9*, ***HTR3A***, ***KCNK13*** *, MAPT*, ***SCN4A***	2.90E‐02	*APP, CHCHD10, MAPT, PSEN1, VCP*	NA	NA

Abbreviations: ADSP, Alzheimer's Disease Sequencing Project; FDR, false discovery rate; GWAS, genome‐wide association study; NA, not applicable.

Genes unique to the ADSP+ list are shown in bold font. Complete results for the ADSP+ (Table 2), familial dementia (Table S2), and GWAS (Table S3) lists are provided in supporting information.

The ADSP+ pathway analyses identified significant enrichment of 33 GO Biological Processes that were not significantly enriched in either the familial dementia or AD GWAS pathway analyses (Table 2). Among these, maintenance of location in cell (GO:0051651; *AKAP9, APOE, GPAA1*), positive regulation of microtubule polymerization (GO:0031116; *AKAP9, MAPT*), and negative regulation of macroautophagy (GO:0016242; *NPC1, SCFD1*) share the strongest evidence of enrichment among the pathways (FDR = 0.0026). Glial cell development (GO:0021782; FDR = 5.70E‐10) and regulation of Aβ formation (GO:1902003; FDR = 3.80E‐12) were the most significantly enriched biological processes in the familial dementia and AD GWAS gene sets, respectively.

**FIGURE 2:**
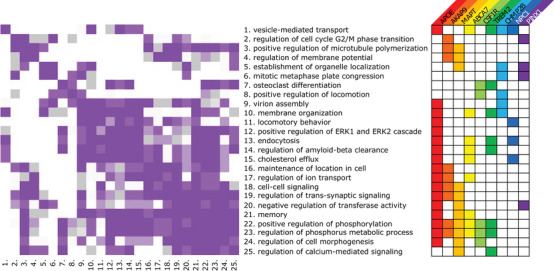
Heatmap of relationships between pathways implicated by Alzheimer's Disease Sequencing Project (ADSP)+ pathway analysis. Left: matrix of pathways significantly enriched in members of the ADSP+ gene set (false discovery rate [FDR] < 0.05) which involve the genes with broadest membership across the ADSP+ pathways. Fisher's exact tests were used to test for overlap in the genes driving the enrichment of each pathway, with *P*‐value encoded by color: *P *> .01 are shown in white, *P*‐values between 0.05 and 0.1 are shown in gray, and *P*‐values between 0 and 0.05 are purple. The gray and purple values are divided into thirds, with darker colors representing smaller values. Right: Matrix indicating the presence/absence of a listed gene (x‐axis) and a pathway (y‐axis). An extended version of this figure including all 45 pathways implicated by the ADSP+ pathway analysis is available in Figures S1 and S2 in supporting information

Many of the 45 pathways identified in the ADSP+ pathway enrichment analysis share contributing genes: 21 pathways involve *APOE*, 12 pathways involve *AKAP9* and/or *MAPT*, 10 pathways involve *ABCA7*, and 9 pathways involve *CSF1R* and/or *TREM2* (Table 2). The right matrix in Figure 2 summarizes contribution of each of these genes to each pathway, while the left matrix illustrates the evidence for significant overlap between the genes driving enrichment of each pathway, where *P *< .05 is shown in purple (Figure 2, Figures S1 and S2 in supporting information). *APOE*, *AKAP9*, and *MAPT* are involved in 30/45 ADSP+ enriched biological processes. Across the most frequent ADSP+ contributors to pathway enrichment, *AKAP9* is the only gene absent from the familial dementia and AD GWAS gene sets. *AKAP9* appears in 12 ADSP+ enriched pathways, second only to *APOE*. The genes contributing to the enrichment of 277 of 990 pairs of pathways implicated by the ADSP+ gene set significantly overlap (*P* < .05). As expected, some pairs of pathways describe similar functions (e.g., positive regulation of phosphorus metabolic process and regulation of phosphorus metabolic process). However, other pairs of pathways share similar genetic profiles yet may implicate distinct mechanisms for AD pathogenesis (e.g., membrane organization and endocytosis).

## 4 DISCUSSION

While the genetic architecture and etiology of AD remains only partially understood, our structured literature review and gene set enrichment analyses suggest that WGS and WES studies may fill in some of these gaps while also providing support for pathways previously implicated in AD. Although each gene set provided a long list of candidate genes with few overlapping genes, the ADSP+ gene set was enriched in biological processes also implicated by the familial dementia genes, AD GWAS genes, or both. This suggests the alternative strategies used to associate these genes with AD point to shared mechanisms of disease.

The presence of pathways associated with regulation of Aβ clearance, endocytosis, regulation of phosphorous metabolic process, immune system process, and regulation of MAPK cascade in all three gene sets support candidate and gene pathways nominated by AD GWAS.^36^, ^37^, ^38^ The relationship between regulation of Aβ clearance (GO:1900221) and cholesterol efflux (GO:0033344) pathways and AD are well established.^39^, ^40^ The regulation of Aβ clearance is directly related to the hallmark pathologic features of AD and offers a connection between the genes implicated in late‐onset AD^41^ and ADAD. Similarly, the relationship between cholesterol efflux and AD has been of interest since the association between *APOE* and AD was first reported.^11^ The ADSP+ studies also provide unique genes to these commonly implicated pathways, further elucidating the mechanisms by which these pathways contribute to the progression of AD.

Among the pathways significantly enriched only by the ADSP+ gene set, one of the most strongly associated processes is positive regulation of microtubule polymerization (GO:0031116; FDR = 0.0026; *AKAP9* and *MAPT*; Table 2). Microtubule polymerization events play important roles in synaptic plasticity and function,^42^ biological processes highlighted by a recent family‐based WGS study of AD.^43^ Tau stabilizes microtubule polymerization, promoting microtubule assembly,^44^ and neurofibrillary tangles of tau are another hallmark of AD pathology.^1^ Post‐translational modifications of tau are known to contribute to neurodegenerative aggregation and affect the ability of tau to promote microtubule polymerization.^45^ Microtubule deficiencies in brain tissue are significantly associated with clinical AD status,^46^ and variation at the *MAPT* locus has been associated with AD among *APOE* ε4 negative subjects.^47^

Although *AKAP9* is specific to the ADSP+ gene set in this study, it was evaluated by the ADSP as a candidate gene with prior evidence of association with AD.^26^ Other AD sequencing studies have identified rare variants with large effect sizes in *AKAP9*,^17^, ^48^ and variants in *AKAP9* were nominally associated with AD in a recent GWAS of African American samples.^14^ *AKAP9* mutations enhance phosphorylation of tau,^49^ directly influence the development of neurofibrillary tangles,^17^ and the gene is upregulated in the hippocampi of patients in early stages of AD.^49^ Among the ADSP+ enriched pathways, *AKAP9* often appears alongside *APOE* and *MAPT* in pathways including cell–cell signaling (GO:0007267), positive regulation of phosphorylation (GO:0042327), regulation of phosphorous metabolic process (GO:0051174), and regulation of trans‐synaptic signaling (GO:0099177). These pathways echo results from a recent study using Bayesian networks to model relationships between epigenomic and transcriptomic data to identify AD networks, where protein phosphorylation and synaptic signaling were identified as differential subnetworks associated with AD.^50^

We have shown that large‐scale sequencing studies like the ADSP bring attention to new genes and biological processes implicated in AD while providing support for biological processes previously nominated by GWAS and family studies. Furthermore, the frequency with which *AKAP9* contributed to both new and established AD pathways and evidence from functional studies that it relates to tau‐mediated AD pathology strengthens the evidence it may play a role in AD risk and pathogenesis.

Our study has several limitations. The ADSP study design included a complicated ascertainment strategy, favoring families with many cases and few *APOE* ε4 alleles, while age, sex, and *APOE* genotype were used to select cases and controls with reduced risk of developing AD.^20^ The sample size of the ADSP Discovery Phase was much smaller than the large‐scale GWAS conducted in recent years.^12^, ^13^ The WGS data in the ADSP Discovery Phase was limited to hundreds of samples representing fewer families; as most AD GWAS signals fall outside of the exome, this may partially explain the minimal overlap between the ADSP+ and GWAS gene sets. It is also important to note that many of the studies that contributed samples to the ADSP are also represented in other AD genetics studies, meaning some samples contribute to both ADSP and GWAS publications. The ADSP Follow‐up study is generating WGS data for thousands of additional subjects with a focus on diverse populations. This increase in diversity and sample size in WES/WGS analyses may provide further insights into the complex genetic architecture of AD. Our analytical approach also has its own limitations. The gene or genes underlying a GWAS or linkage signal are not always clear; gene sets prioritizing different genes within these loci may implicate different pathways. Gene sets which include genes implicated by studies of AD endophenotypes, biomarkers, or studies better representing non‐European ancestry may also implicate additional pathways in AD. While gene set enrichment analysis is a useful tool for providing biological context for genes, there is no single gold‐standard approach. This study focused on GO: Biological Processes, as our approach accounted for the ontological relationships between processes and this approach has been widely used in AD genetics studies (e.g., Jansen *et al*.^12^ and Kunkle *et al*.^13^). GO: Biological Processes have complex relationships and can be broadly defined; alternative pathway analysis strategies using a different source for pathway definitions or requiring a different number of genes to contribute to an enrichment signal will yield different results. Despite the limitations, gene set analysis and other pathway analysis tools provide a mechanism of hypothesis generation for disease susceptibility.

## ADSP BANNER AUTHOR LIST

Members of the Discovery Phase of the Alzheimer's Disease Sequencing Project included: Michelle Bellair, Huyen Dinh, Harsha Doddapeneni, Shannon Dugan‐Perez, Adam English, Richard A Gibbs, Yi Han, Jianhong Hu, Joy Jayaseelan, Divya Kalra, Ziad Khan, Viktoriya Korchina, Sandra Lee, Yue Liu, Xiuping Liu, Donna Muzny, Waleed Nasser, William Salerno, Jireh Santibanez, Evette Skinner, Simon White, Kim Worley, Yiming Zhu, Alexa Beiser, Yuning Chen, Jaeyoon Chung, L Adrienne Cupples, Anita DeStefano, Josee Dupuis, John Farrell, Lindsay Farrer, Daniel Lancour, Honghuang Lin, Ching Ti Liu, Kathy Lunetta, Yiyi Ma, Devanshi Patel, Chloe Sarnowski, Claudia Satizabal, Sudha Seshadri, Fangui Jenny Sun, Xiaoling Zhang, Seung Hoan Choi, Eric Banks, Stacey Gabriel, Namrata Gupta, William Bush, Mariusz Butkiewicz, Jonathan Haines, Sandra Smieszek, Yeunjoo Song, Sandra Barral, Phillip L. De Jager, Richard Mayeux, Christiane Reitz, Dolly Reyes, Giuseppe Tosto, Badri Vardarajan, Shahzad Amad, Najaf Amin, M Afran Ikram, Sven van der Lee, Cornelia van Duijn, Ashley Vanderspek, Helena Schmidt, Reinhold Schmidt, Alison Goate, Manav Kapoor, Edoardo Marcora, Alan E. Renton, Kelley Faber, Tatiana Foroud, Michael Feolo, Adam Stine, Lenore J. Launer, David A. Bennett, Li Charlie Xia, Gary Beecham, Kara Hamilton‐Nelson, James Jaworski, Brian Kunkle, Eden Martin, Margaret Pericak‐Vance, Farid Rajabli, Michael Schmidt, Thomas H. Mosley, Laura Cantwell, Micah Childress, Yi‐Fan Chou, Rebecca Cweibel, Prabhakaran Gangadharan, Amanda Kuzma, Yuk Yee Leung, Han‐Jen Lin, John Malamon, Elisabeth Mlynarski, Adam Naj, Liming Qu, Gerard Schellenberg, Otto Valladares, Li‐San Wang, Weixin Wang, Nancy Zhang, Jennifer E. Below, Eric Boerwinkle, Jan Bressler, Myriam Fornage, Xueqiu Jian, Xiaoming Liu, Joshua C. Bis, Elizabeth Blue, Lisa Brown, Tyler Day, Michael Dorschner, Andrea R. Horimoto, Rafael Nafikov, Alejandro Q. Nato Jr., Pat Navas, Hiep Nguyen, Bruce Psaty, Kenneth Rice, Mohamad Saad, Harkirat Sohi, Timothy Thornton, Debby Tsuang, Bowen Wang, Ellen Wijsman, Daniela Witten, Lucinda Antonacci‐Fulton, Elizabeth Appelbaum, Carlos Cruchaga, Robert S. Fulton, Daniel C. Koboldt, David E. Larson, Jason Waligorski, Richard K. Wilson.

## Supporting information



Supporting Information

Supporting Information

Supporting Information
