# Supplementary material for: Large‐scale sequencing studies expand the known genetic architecture of Alzheimer's disease
Source: Alzheimers Dement (Amst). 2021 Dec 31;13(1):e12255. doi: 10.1002/dad2.12255 (PMC8720139; doi:10.1002/dad2.12255)
Supplement: Supplementary file 2 — Supporting Information [file DAD2-13-e12255-s003.pdf]

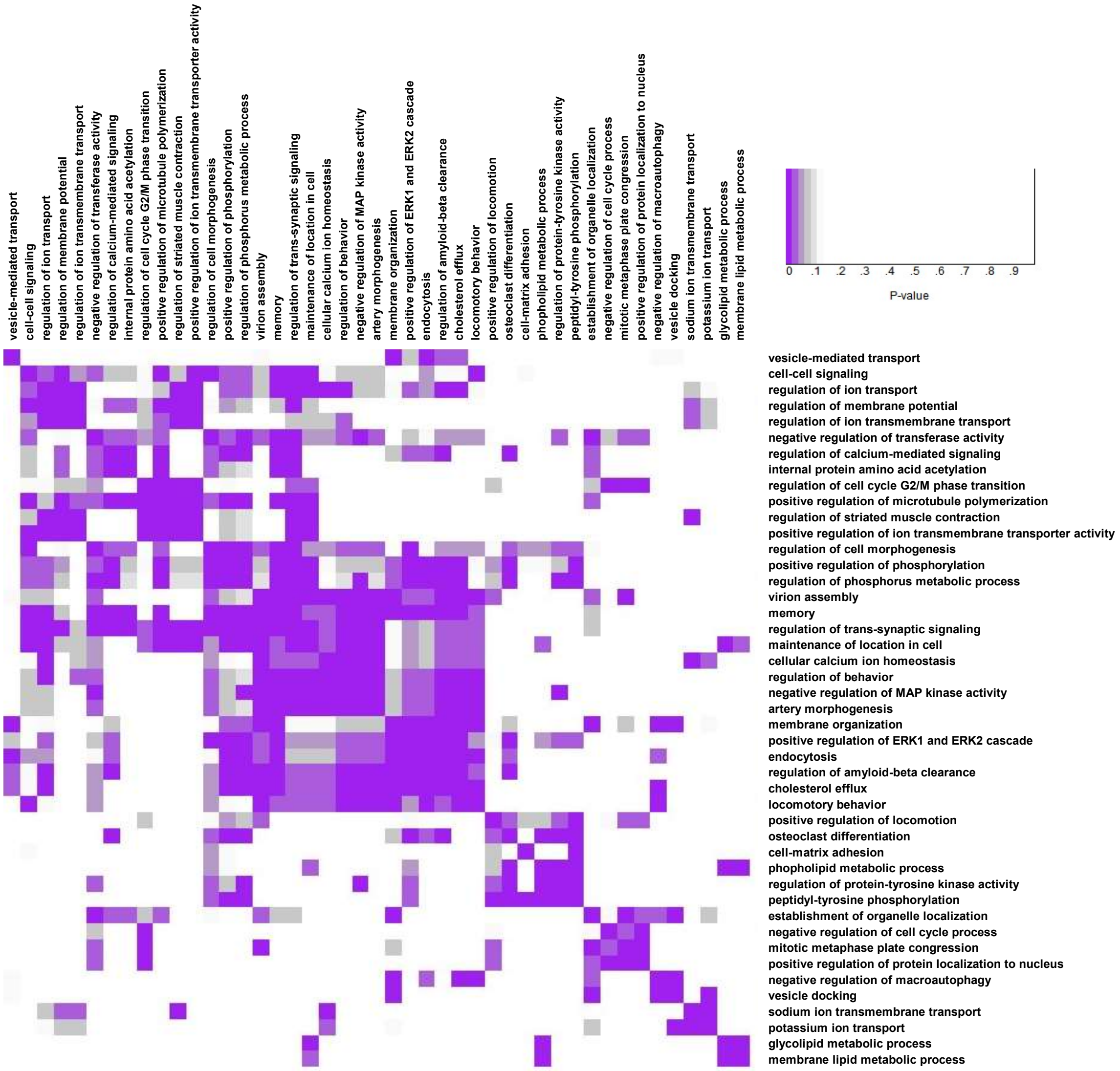

Supplemental Figure 1: Heatmap of relationships between all pathways implicated by ADSP+ pathway analysis. Matrix of pathways significantly enriched in members of the ADSP+ gene set (False Discovery Rate, FDR < 0.05). Fisher's Exact tests were used to test for overlap in the genes driving the enrichment of each pathway, with P < 0.05 shown in purple.
