# Supplementary material for: Large‐scale sequencing studies expand the known genetic architecture of Alzheimer's disease
Source: Alzheimers Dement (Amst). 2021 Dec 31;13(1):e12255. doi: 10.1002/dad2.12255 (PMC8720139; doi:10.1002/dad2.12255)
Supplement: Supplementary file 3 — Supporting Information [file DAD2-13-e12255-s001.pdf]

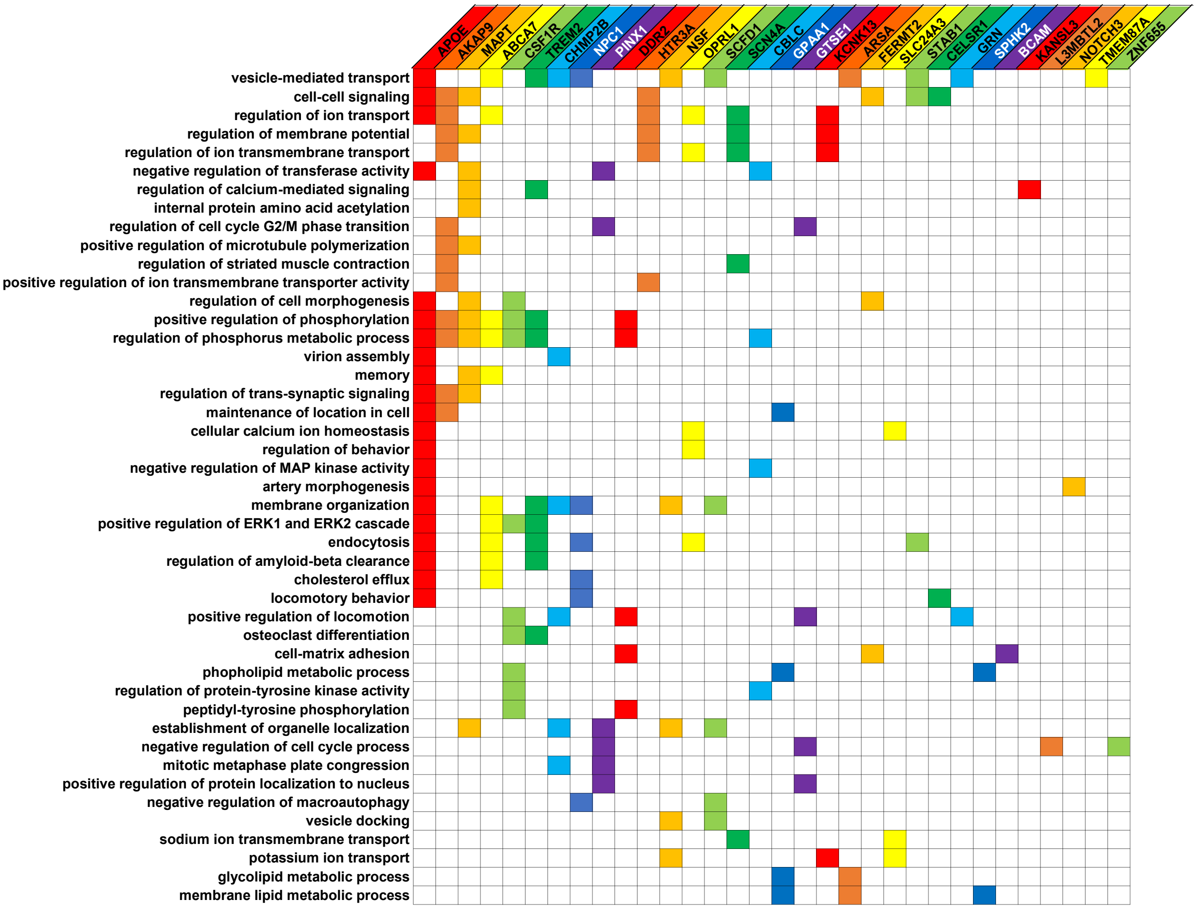

Supplemental Figure 2: Relationships between genes and enriched pathways in the ADSP+ analysis. Matrix indicating the presence/absence of a listed gene (x-axis) and a pathway (y-axis).
